# SLIMM: species level identification of microorganisms from metagenomes

**DOI:** 10.7717/peerj.3138

**Published:** 2017-03-28

**Authors:** Temesgen Hailemariam Dadi, Bernhard Y. Renard, Lothar H. Wieler, Torsten Semmler, Knut Reinert

**Affiliations:** 1 Department of Mathematics and Computer Science, Freie Universität Berlin, Berlin, Germany; 2 International Max Planck Research School for Computational Biology and Scientific Computing (IMPRS-CBSC), Berlin, Germany; 3 Department of Veterinary Medicine, Freie Universität Berlin, Berlin, Germany; 4 Robert Koch Institute, Berlin, Germany; 5 Max Planck Institute for Molecular Genetics, Berlin, Germany

**Keywords:** Metagenomics, Microbial communities, Microorganisms, Taxonomic profiling, NGS data, Microbiology

## Abstract

Identification and quantification of microorganisms is a significant step in studying the alpha and beta diversities within and between microbial communities respectively. Both identification and quantification of a given microbial community can be carried out using whole genome shotgun sequences with less bias than when using 16S-rDNA sequences. However, shared regions of DNA among reference genomes and taxonomic units pose a significant challenge in assigning reads correctly to their true origins. The existing microbial community profiling tools commonly deal with this problem by either preparing signature-based unique references or assigning an ambiguous read to its least common ancestor in a taxonomic tree. The former method is limited to making use of the reads which can be mapped to the curated regions, while the latter suffer from the lack of uniquely mapped reads at lower (more specific) taxonomic ranks. Moreover, even if the tools exhibited good performance in calling the organisms present in a sample, there is still room for improvement in determining the correct relative abundance of the organisms. We present a new method Species Level Identification of Microorganisms from Metagenomes (SLIMM) which addresses the above issues by using coverage information of reference genomes to remove unlikely genomes from the analysis and subsequently gain more uniquely mapped reads to assign at lower ranks of a taxonomic tree. SLIMM is based on a few, seemingly easy steps which when combined create a tool that outperforms state-of-the-art tools in run-time and memory usage while being on par or better in computing quantitative and qualitative information at species-level.

## Introduction

In the context of microbial communities, alpha diversity is the mean diversity of a single microbial community and one way to represent diversity (richness) is using the number of different species in a given sample. Beta diversity on the other hand is the degree to which the species composition of the various microbial communities differ from another (Whittaker, 1960). Determining the alpha and beta diversity of microbial communities in relevance to the host corresponding environment is ubiquitous in comparative metagenomics. Due to this identification and quantification of microorganisms using shotgun metagenomic reads obtained by Next Generation Sequencing (NGS) has become a subject of growing interest in the field of microbiology. The publication of numerous taxonomic profiling tools within the last decade only shows how appealing the subject truly is. Lindgreen, Adair & Gardner (2016) considered 14 different sequence classification tools based on various approaches in a recent review of such methods.

Turning raw metagenomic reads into the relative abundance of multiple groups of microorganisms (clades) residing on the sample from which the environmental DNA was extracted and sequenced is a complicated task for several reasons. To mention a few: (1) shared (homologous) regions of genome sequences across multiple microorganisms make an assignment of reads to their true exact difficult. (2) The range of variation in the abundance of individual groups of microbes in the sample can be high. In such cases, it is harder to detect the least abundant ones and not mistake them for noise. (3) The high degree of variation in publicly available genome sequence lengths of different microbes makes the quantification non-trivial (Brady & Salzberg, 2009).

In the past benchmarking of taxonomic profiling tools was done at the genus or higher level of the taxonomic tree. This is due to the shortcomings of many earlier tools to report species-level taxonomic profiles with acceptable accuracy. However, species-level resolution of microbial communities is desirable and more modern tools do address this (Lindgreen, Adair & Gardner, 2016; Piro, Lindner & Renard, 2016; Lindner & Renard, 2015; Francis et al., 2013). For this reason, all the benchmarks in this study were done at species-level.

In general, two distinct approaches have been widely used to tackle the challenge of ambiguous reads that originate from genomic locations shared among multiple groups of organisms. The first approach is to prepare a signature-based database with sequences that are unique to a clade. This method represents taxonomic clades uniquely by sequences that do not share common regions with other clades of the same taxonomic rank. Even if this approach makes use of the fraction of metagenomic data from the sequencer, it can guarantee to have only a single assignment of sequencing reads to a clade. Tools like MetaPhlAn2 (Truong et al., 2015), GOTTCHA (Freitas et al., 2015) and mOTUs (Sunagawa et al., 2013) use this method. The second approach is based on using the full set of reference sequences available as a database and assigning ambiguous reads to their least common ancestor (LCA) in a taxonomic tree. Kraken (Wood & Salzberg, 2014), a k-mer based read binning method, is an example of such an approach. Both approaches have certain advantages and disadvantages. The former has an advantage in speed and precision but is limited to utilizing the reads that can be mapped uniquely to the curated regions. The latter approach, on the other hand, suffers from the lack of uniquely mapped reads at lower (more specific) taxonomic ranks.

Based on the final output of a method there are two categories of metagenomic classification tools i.e., a read binning method and a taxonomic profiling method. A read binning method assigns every single read to a node in a taxonomic tree, whereas a taxonomic profiling method tries to report which organisms or clades are present in the sample with or without having to assign every read to a corresponding taxon. There exists an overlap between the two categories making it possible for some read binning methods to be used as a taxonomic profiling tool as well.

GOTTCHA uses a signature-based database specific to a given taxonomic rank, and it is highly optimized for low false discovery rate (FDR). Kraken instead uses a database comprising a hash table of k-mers and their corresponding node in a given taxonomic tree. Then it assigns reads based on where the majority of its k-mers are located in the tree. Whenever no clear vote by the k-mers of the read exists, Kraken will assign that read to its least common ancestor. Kraken is a very fast read binning method, which is also often used to do taxonomic profiling. mOTUs uses single copy universal marker genes to achieve a species-level abundance resolution of microbial communities. Even if the tools exhibited good performance in calling the organisms present in a sample, there is still room for improvement in determining the correct relative abundance of the detected organisms.

In the following, we present a novel method called **S**pecies **L**evel **I**dentification of **M**icroorganisms from **M**etagenomes (**SLIMM**), which addresses the limitations noted above. During the preprocessing stage, we gather from a group of interests (e.g., Archaea, Bacteria, Viruses or any combination of these) as many reference sequences as possible and downsize and compile taxonomic information of the gathered sequences. The taxonomic information is stored in the form of the SLIMM database (SLIMM_DB). We then use a read mapper to align metagenomic reads against the gathered reference sequences, which we consider as a preprocessing step that is often done for numerous other analyses as well (we will report the run time and memory requirement with and without preprocessing). SLIMM works on the resulting BAM/SAM alignment file. First, SLIMM uses coverage information both by the reads that mapped on different reference sequences and by reads uniquely mapped to a reference sequence to remove unlikely genomes from the analysis similar to an approach taken by Lindner et al. (2013). This filtration, in turn, allows us to subsequently gain a larger number of uniquely mapped reads assigned to the reduced set of genomes which we can assign to lower ranks of a taxonomic tree. We will show that this simple approach has indeed positive effects on the analysis. The second step is to assign the remaining non-uniquely mapped reads to the lowest common ancestor. Overall SLIMM is based on a few, seemingly easy steps resulting in a tool that outperforms state-of-the-art tools in run-time and memory usage while being on par or better in computing quantitative and qualitative information at the species-level which we show in the results section. Following the recommendation in Piro, Lindner & Renard (2016) with caution, we have carried out digital normalization on the raw reads (Brown et al., 2012) which discards low quality and redundant reads. This works by removing reads belonging to a region with high coverage depth. In our experience, the digital normalization showed a negligible improvement in calling the correct organisms.

## Method

### Nonredundant Reference genomes database

Reference genomes from NCBI GenBank (ftp://ftp.ncbi.nlm.nih.gov/genomes/genbank/) and RefSeq (ftp://ftp.ncbi.nlm.nih.gov/genomes/refseq) archives, downloaded on 21.05.2016, were used for the method described here. SLIMM is not limited to these public databases when provided a proper mapping from sequence identifiers to a taxonomic id and a taxonomic tree that represents all the sequences in the database. For this study, we considered microbes under the super-kingdom of archaea and bacteria. However, one can also easily integrate viruses into the database by using the provided SLIMM preprocessing tool. Before downloading all the genomes, we checked for redundancy by counting the number of available files for each species of interest. If multiple genomes were available for download, we then chose one in the order of (1) RefSeq (2) Complete Genome and (3) Draft Genome. This way, we received as many species as possible represented by their best reference genome so far available. After downloading the sequences, we checked if every genomic file contained only a single FASTA entry. If not, we take their concatenation separated by a contiguous sequence of ten N’s so that reads will not accidentally map at the joining point. The final result is a reference genome library of organisms from the interest groups, which contains a single representative sequence per species. To cope with the dynamically expanding reference genomes library, we implemented a feature for the SLIMM preprocessing tool that can seamlessly update the reference genome database. In this way, we received two databases that we named small_DB and large_DB. Small_DB contains 2163 species with their corresponding complete genomes while large_DB contains 13,192 species including those with only draft genomes available.

### Read mapping against a database of interest

**Figure 1:**
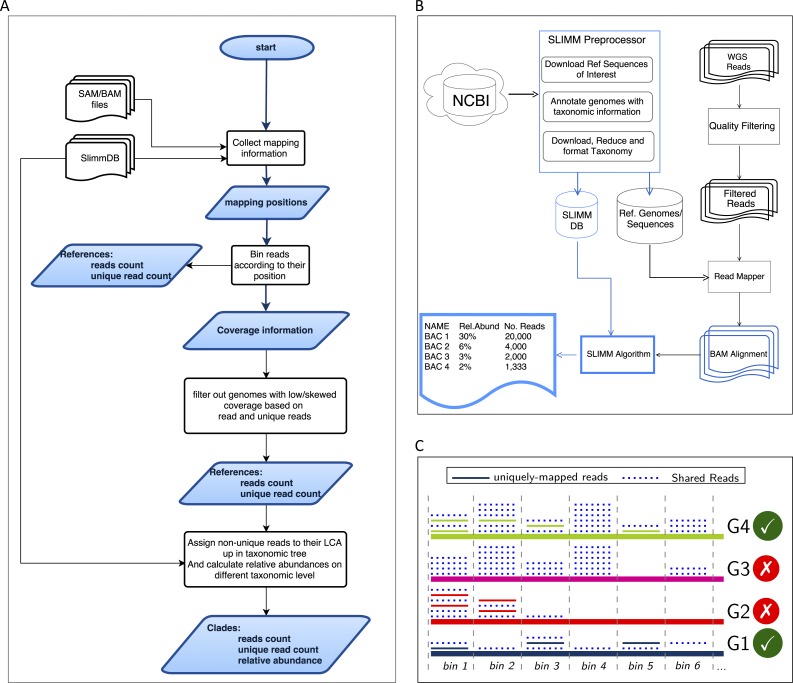
Overview of the SLIMM methodology: (A) The SLIMM algorithm: SLIMM takes two inputs, i.e., the SLIMMDB and an alignment file in either SAM or BAM format and calculates statistical data for each reference sequences in the database. SLIMM uses coverage information to leave out reference sequences from consideration and recalculate the statistics again. We use this, in turn, to receive read counts that are uniquely mapped to a clade at a given taxonomic rank. (B) SLIMM Pipeline: the preprocessing module of SLIMM downloads/updates all available genomes of a certain interest group (e.g., Archaea, Bacteria, Viruses or any combination of them) and tags the sequences with their corresponding taxonomic information. A read mapper is then used to map the WGS reads to these reference sequences. Then SLIMM algorithm uses the mapping results to produces taxonomic profile reports. (C) Reference filtering based on coverage information: an illustration of how SLIMM uses reference filtering based on coverage information: G2 and G3 could not pass the filtering steps because they did not contain enough coverage by uniquely mapped reads and all reads respectively.

SLIMM requires an alignment/mapping file in SAM or BAM format as an input (Fig. 1A). The alignment file can be obtained by aligning the short metagenome shotgun sequencing reads against a library of reference genomes of interest (Fig. 1B). To do so, one can use a read mapper of choice. Nevertheless, the pipeline could benefit from a faster but yet accurate read mapper as this preprocessing step is relatively time-consuming. We make the read mapping program output secondary alignments because (1) it is very likely to have a sequencing read mapped to multiple targets, (2) a read might have multiple best hits and (3) the best hit of a read might not be its true origin. SLIMM uses coverage landscape information as shown in Fig. 1C to resolve this. We used bowtie2 (Langmead & Salzberg, 2012) and Yara (Siragusa, 2013) in our preliminary experiments because they are known to be fast read mappers with multi-threading options. Since Yara is several times faster, does not employ heuristics and its resulting alignments produced better profiles in some of the cases, we used it as the default mapper for this study.

### Collecting coverage information of each reference genome

We first identify which reads are mapped to which reference genomes. Then we separate the reads uniquely assigned to a single reference sequence from those assigned to multiple reference sequences. Reads that are mapped to multiple places within a reference are also considered uniquely mapped. During this stage, SLIMM collects information like the number of reference genomes with mapping reads, the total number of reads and the average read length, which will later be used for discarding reference genomes. We then map reads into bins of specific width across each reference genome based on the location of their mapping. The binning is done twice, once for mapped reads in general and once only for uniquely mapped reads. The default width for the bins is set to the average length of sequencing reads with an option to set it to a different value. Higher bin width means fewer bins and faster runtime, but it could lead to underrepresentation of coverage information which in turn is based on whether a bin is empty or not. The bin number corresponding to a read mapped to a reference is defined by the centeral position of its mapping location divided by the width of the bins (integral part only). The bin number of a read mapped to a reference starting from *loc*_*start*_ all the way to *loc*_*end*_ is given by:

(1) \begin{eqnarray*}binNumber= \left\lfloor \frac{lo{c}_{start}+lo{c}_{end}}{2\times w} \right\rfloor \end{eqnarray*}

where *w* is the width of bins a reference is partitioned into.

After binning is done, coverages based on mapping reads and uniquely mapped reads are calculated based on the corresponding bin sets. Coverage information of each reference sequence is represented by coverage percentage (*%Cov*) and coverage depth (*CovDepth*) as shown in Eqs. (2) and (3) respectively.

(2) \begin{eqnarray*}& & \text{%}Cov= \frac{{|}nonzeroBins{|}}{{|}bins{|}} \times 100\end{eqnarray*}

(3) \begin{eqnarray*}& & \text{CovDepth}= \frac{\sum _{i=1}^{{|}bins{|}}\sum _{j=1}^{{N}_{bin}}readLength}{{|}bins{|}} \end{eqnarray*}

Where |*nonzeroBins*| is the number of non-zero bins, |*bins*| is the total number of bins in the reference, *N*_*bin*_ is the number of reads in a bin and *readLength* is the number of bases in a read.

### Discarding unlikely genomes based on coverage landscape

We discard reference sequences with coverage percentages below a specific threshold. The threshold is calculated based on a given percentile (default 0.001) of all coverage percentages of the genomes. In other words, after sorting the reference sequences based on their coverage percentages in descending order we take the top N sequences that cover 99.999% of the sum of all coverage percentages. This step is done for both coverage percentage by reads that mapped on multiple references and uniquely mapped reads. This process eliminates many genomes even if they have a lot of reads mapping to them as long as they do not have a good enough coverage. Furthermore, this method was also proven to eliminate reference sequences that acquire a stack of reads only in one or two bins across their genomes which could be a result of either a sequencing artifact or a conserved region in the genome among distant relatives.

### Recalculating reads uniqueness after discarding unlikely genomes

After discarding reference sequences, SLIMM recalculates the uniqueness of the reads again. This recalculation can increase the number of uniquely mapped reads assigned to lower-level clades in a taxonomic tree. The recalculation of uniquely mapped reads is shown to improve the abundance estimation of a clade.

### Assigning reads to their LCA and calculating abundances at a given rank

After recalculating the uniqueness of reads, we assign non-uniquely mapped reads to their LCA taxon based on the NCBI taxonomic tree downloaded from ftp://ftp.ncbi.nih.gov/pub/taxonomy. Instead of using the whole NCBI taxonomic tree we use a reduced subtree produced by the SLIMM preprocessing tool. Since we only report for a given major taxonomic ranks namely superkingdom (domain), phylum, class, order, family, genus and species, the reduced tree contains only these taxonomic ranks. We also discarded the branches of the tree which are outside of the interest groups i.e., Archaea and Bacteria for this study. This reduction saves a significant amount of computational time as assigning a read to its LCA is computationally expensive. We also propagate the number of uniquely mapped reads at a node to any of its ancestors. Then we calculate the relative abundance of each taxonomic unit at a given rank as the uniquely mapped reads that are assigned to it divided by the total number of uniquely mapped reads at the rank Eq. (4). We also report an aggregated coverage depth of each clade defined as in Eq. (5).

(4) \begin{eqnarray*}& & RelA{B}_{clade}= \frac{{N}_{clade}}{{N}_{mapped}} \end{eqnarray*}

(5) \begin{eqnarray*}& & CovDept{h}_{clade}= \frac{\sum _{i=1}^{{N}_{clade}}readLength}{\sum _{i=1}^{{N}_{child}}refLength} \end{eqnarray*}

where *RelAB*_*clade*_ is the relative abundance of a clade, *N*_*clade*_ is the number of reads that are assigned to a clade, *N*_*mapped*_ is the total number of reads that are mapped to any clade, *CovDepth*_*clade*_ is coverage depth of a clade, *readLength* is the number of bases in a read, and ${\mathop{\sum }\nolimits }_{i=1}^{{N}_{child}}refLength$ is the sum of reference lengths of children of a clade that contribute at least one read.

## Results and Discussion

### Datasets

For this study, we assembled 18 different metagenomic datasets of various origins and simulation strategies. The datasets contain (1) mock community metagenomes from two different studies which were are sequenced using Illumina Genome Analyzer II (2) simulated metagenomes that resemble community profile of a real metagenome as identified by MetaPhlAn2 (Truong et al., 2015) (3) simulation of randomly created microbial communities with a varying number of organisms and range of relative abundances. We used NeSSM (Jia et al., 2013) to do the simulations. (4) Medium complexity CAMI (The Critical Assessment of Metagenome Interpretation) challenge toy datasets that are publicly available at https://data.cami-challenge.org/participate. We believe that this collection of datasets can represent most of the metagenomic communities that a taxonomic identifier will have to handle.

We used three mock community datasets, two from the Human Microbiome Project (HMP) (HMP, 2012) containing genomes of 22 microorganisms and one from the study (Shakya et al., 2013) containing genomes of 64 microorganisms. The two datasets from HMP are similar in the species they contain. They only differ in the abundance distribution. One contains an even abundance distribution of the microorganisms whereas the other contains a differing abundance distribution of the 22 microorganisms.

For simulated datasets resembling an existing community we chose: (1) a metagenome obtained from the human gut sample during the HMP (2012) (2) a freshwater metagenome dataset from Lake Lanier (Oh et al., 2011). We used MetaPhlAn2 (Truong et al., 2015)—a popular metagenomic profiling tool based on use clade-specific marker genes. Next we used the reported profile as a basis for the simulation.

For randomly created microbiomes, we considered three communities with randomly selected member organisms. The number of organisms in these communities is 50, 200 and 500. We then chose three different ranges of relative abundances i.e., even, [1–100] and [1–1,000]. This provided us with a total of 9 randomly created metagenomes with varying complexity both regarding diversity and in abundance differences. The different settings of metagenomic datasets are important to make sure that the tested methods work with a broad range of input datasets. To resemble an actual metagenome and to make the taxonomic profiling more difficult, we contaminated all the simulated datasets with real world metagenomic reads sequenced by Illumina MiSeq, after removing the reads that could be mapped to any of the prokaryotic genomes available. Details of all the datasets used for evaluation can be found in the Supplemental Information.

### Performance comparison

We compared the runtime and accuracy of SLIMM with other existing taxonomic profiling tools. For this we considered GOTTCHA, mOTUs, and Kraken as recent and frequently used reference-based shotgun metagenome classification tools for comparison. For Kraken, we created a Kraken database corresponding to both small_DB and large_DB. We used large_DB only for the CAMI datasets as these datasets contain species for which only draft genomes were available. GOTTCHA and mOTUs use their own special curated database. Table 1 shows the average runtime and the average peak memory usage of the tools across runs on the 14 different datasets, excluding the CAMI datasets, used in this study. We used a machine with 32 (Intel(R) Xeon(R) CPU 3.30 GHz) processors and 378GB of memory. The CAMI datasets are not included in the runtime and memory comparison. That is because we could not ran Kraken with large_DB on the same machine since it required 500GB of memory. Instead, we run Kraken on a cluster for these particular datasets. Without the time needed for the preprocessing SLIMM is proven to be faster than any of the other tools considered while using a fair amount of memory footprint. With the preprocessing, Kraken is faster but uses much more memory. SLIMM is faster than GOTTCHA and mOTUs. More information regarding runtime can be found in the supplement.

**Table 1:** Runtime and memory comparison of SLIMM against existing methods.

	Alignment + SLIMM	Kraken	GOTTCHA	mOTUs
Avg. Runtime (Seconds)	*422.1*+ 61.0	157.4	1727.1	1526.6
Peak Memory (GB)	*33.67*+ 5.2	102	4	1.6

We used different accuracy measures namely precision(specificity), recall (sensitivity) and F1-Score to compare the accuracy of each tool with SLIMM. The definition of the accuracy measures is given below.

(6) \begin{eqnarray*}& & precision= \frac{TP}{TP+FP} \end{eqnarray*}

(7) \begin{eqnarray*}& & recall= \frac{TP}{TP+FN} \end{eqnarray*}

(8) \begin{eqnarray*}& & {F}_{1}=2\times \frac{precision\times recall}{precision+recall} \end{eqnarray*}

where *TP* = true positives (species which are in the samples and called by the tools); *TN* = true negatives (species which are not in the samples and not called by the tools); *FP* = false positives (species which are not in the samples and yet called by the tools) and *FN* = false negatives (species which are in the samples but not called by the tools)

Table 2 shows the results of the performance comparison among SLIMM and existing metagenomic classifiers using 18 different datasets described above. SLIMM outperforms all of the tools in 13 of the 18 cases in precision. SLIMM and Kraken showed good results in recall. SLIMM came in second place exceeding Kraken occasionally. However, Kraken produced a higher number of false positives to attain this recall, hence the lower numbers in precision. GOTTCHA performed well with the HMP datasets while it underperformed in the rest of the datasets in general. mOTUs does not perform well in all of the datasets. We provided F1-Score in the table as a measure of the right balance between precision and recall. SLIMM outperforms all the other tools both in precision and F1-Score in 17 of the 18 cases while Kraken is slightly better in recall for the majority of the cases.

**Table 2:** Comparison of SLIMM against different tools regarding precision and recall on species-level: The highest values in each row are marked bold for both precision and recall.

Precision						Recall				F1			
Type	Dataset	SLIMM	Kraken	GOTTCHA	mOTUs	SLIMM	Kraken	GOTTCHA	mOTUs	SLIMM	Kraken	GOTTCHA	mOTUs
Mock	MG01	0.8923	0.6264	0.9808	**1.0000**	**0.9355**	0.9194	0.8226	0.8065	**0.9134**	0.7451	0.8947	0.8929
	MG02	0.9545	0.8400	**1.0000**	**1.0000**	**1.0000**	**1.0000**	0.9524	0.8571	**0.9767**	0.9130	0.9756	0.9231
	MG03	0.9524	0.6897	**1.0000**	**1.0000**	**0.9524**	**0.9524**	0.8571	0.4286	**0.9524**	0.8000	0.9231	0.6000
Mimic.Sim	MG04	**1.0000**	0.4250	0.6000	0.9474	**1.0000**	**1.0000**	0.6176	0.5294	**1.0000**	0.5965	0.6087	0.6792
	MG05	**1.0000**	0.6650	0.8714	0.9630	**1.0000**	**1.0000**	0.4656	0.1985	**1.0000**	0.7988	0.6070	0.3291
Rand.Sim	MG06	**0.9783**	0.4352	0.6897	0.8718	0.9375	**0.9792**	0.8333	0.7083	**0.9574**	0.6026	0.7547	0.7816
	MG07	**0.9783**	0.4352	0.6964	0.9091	0.9375	**0.9792**	0.8125	0.6250	**0.9574**	0.6026	0.7500	0.7407
	MG08	**0.9783**	0.4299	0.7143	0.8824	0.9375	**0.9583**	0.8333	0.6250	**0.9574**	0.5935	0.7692	0.7317
	MG09	**0.9929**	0.7220	0.8396	0.9286	0.9211	**0.9737**	0.5855	0.3421	**0.9556**	0.8291	0.6899	0.5000
	MG10	**0.9930**	0.7178	0.7949	0.9574	0.9276	**0.9539**	0.4079	0.2961	**0.9592**	0.8192	0.5391	0.4523
	MG11	**0.9928**	0.7164	0.8058	0.9464	0.9079	**0.9474**	0.5461	0.3487	**0.9485**	0.8159	0.6510	0.5096
	MG12	**0.9855**	0.8284	0.7333	0.9773	0.9315	**0.9589**	0.0377	0.1473	**0.9577**	0.8889	0.0717	0.2560
	MG13	**0.9855**	0.8237	0.8095	0.9811	**0.9315**	0.9281	0.0582	0.1781	**0.9577**	0.8728	0.1086	0.3014
	MG14	0.9851	**0.9857**	0.8000	0.9811	0.9041	**0.9452**	0.0548	0.1781	0.9429	**0.9650**	0.1026	0.3014
CAMI	MG15	**0.9261**	0.7644	0.7397	0.8000	**0.8191**	0.7990	0.2714^*^	0.1206^*^	**0.8693**	0.7813	0.3971^*^	0.2096^*^
	MG16	0.8377	0.7027	0.6883	**0.8462**	**0.8040**	0.7839	0.2663^*^	0.1106^*^	**0.8205**	0.7411	0.3841^*^	0.1956^*^
	MG17	**0.9302**	0.7608	0.4531	0.7368	**0.8040**	0.7990	0.1457^*^	0.1407^*^	**0.8625**	0.7794	0.2205^*^	0.2363^*^
	MG18	**0.8223**	0.6996	0.4839	0.7778	**0.8141**	0.7839	0.1508^*^	0.1407^*^	**0.8182**	0.7393	0.2299^*^	0.2383^*^

**Notes.**

* GOTTCHA and mOTUs have unfairly lower recall and F1 values due to their database which does not contain the complete set of references for the corresponding datasets.

**Figure 2:**
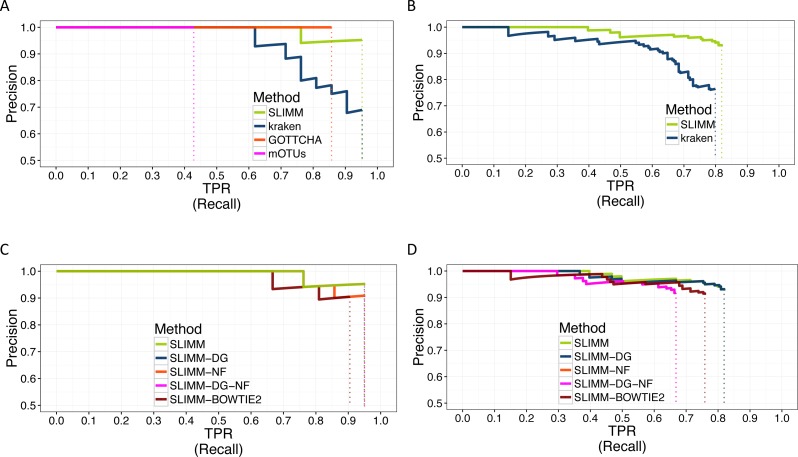
PR Curves: comparison of SLIMM against existing methods (A) and (B): true Positive Rate(TPR)/recall drawn against precision. SLIMM showed the highest performance. GOTTCHA did not discover any false positives but is low in recall. PR curves different variants of SLIMM (C) and (D): SLIMM i.e., SLIMM-DG (with digital normalization), SLIMM-NF (without filtration step based on coverage landscape), SLIMM-NF-DG (without filtration but with digital normalization) and SLIMM using alignment produced by the read mapper Bowtie2.

We did a PR curve analysis for the HMP mock community dataset with uneven distribution of relative abundances of member organisms and one of the CAMI challenge datasets. We sorted the predicted species by predicted abundance in decreasing order to draw the PR curves. The PR curves in Fig. 2 show that SLIMM has a better recall rate than the other tools while staying precise.

**Figure 3:**
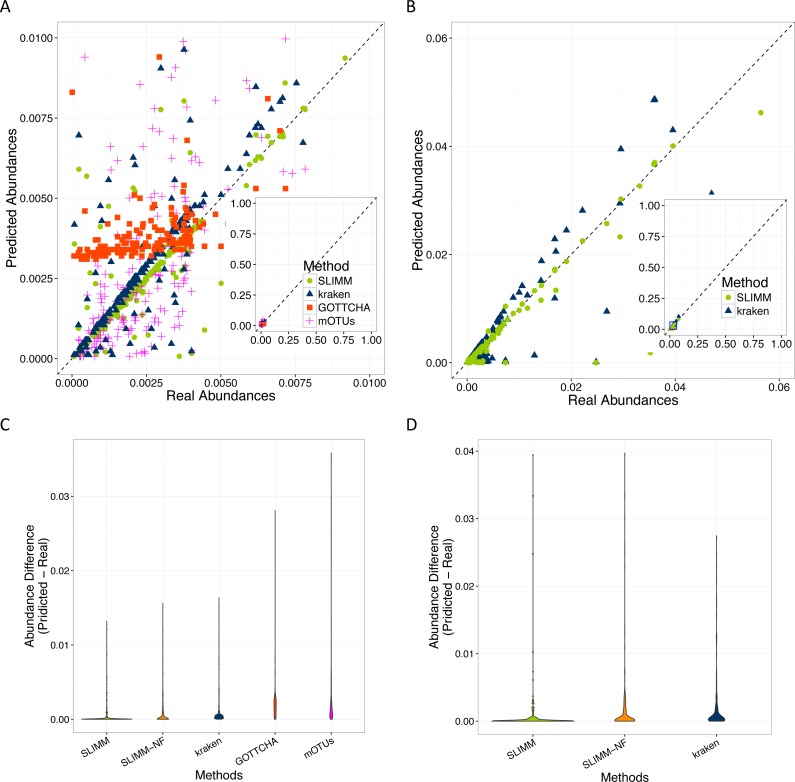
Predicting abundances correctly (A)—Random Dataset and (B)—CAMI Dataset: Abundances predicted by different tools compared to the true abundance used for simulation. SLIMM predicted the abundances more accurately than the other tools. Kraken overestimates the abundance. GOTTCHA and mOTUs did not perform well in predicting the abundances. Violin plots (C)—Random Dataset and (D)—CAMI Dataset: SLIMM has the lowest divergence from true abundances.

SLIMM’s ability to predict the correct abundances of organisms better than the existing methods is visualized by the scatterplots in Figs. 3A and 3B by plotting the true abundance of organisms against their predicted abundance by different tools for one of the CAMI challenge datasets and one of the randomly simulated datasets. From these plots, it can clearly be seen that SLIMM predicts the abundance more accurately. Even though it was not originally developed for abundance estimation, the next best tool is Kraken which slightly overestimates the true abundance. mOTUs and GOTTCHA do not perform well at predicting the abundances.

Violin plots are similar to box plots, but additionally they visualize the density distribution of different data points. The violin plots in Figs. 3C and 3D show how good the different tools predicted the abundances compared to the actual abundances. In these plots, we can see that SLIMM has the lowest divergence from the true abundance. For the randomly simulated dataset, SLIMM has an average absolute difference of *0.00073* and Kraken has an average absolute difference of *0.00116* which is 159% higher compared to SLIMM. For the same dataset, GOTTCHA and mOTUs have an average absolute difference of *0.00206* and *0.00273* respectively. SLIMM also received the most correct (closer) abundances with absolute differences of first quartile (*Q*1) = 0.00002 and third quartile (*Q*3) = 0.00016. Kraken is the second-best tool in this regard with values *Q*1 = 0.00018, *Q*3 = 0.00065.

We have also investigated the positive effects of the filtering step in SLIMM. We ran SLIMM with the filter turned off and compared the results with a standard run of SLIMM. Figures 2C and 2D show that the filtration step overall leads to better results. It is also interesting to note that SLIMM’s filtration step effectively reduces the divergence from the true abundance. Figures 3C and 3D show that SLIMM’s filtration step produced abundances closer to the real one. The quartiles of absolute differences between real and predicted abundances are (*Q*1 = 0.00002, *Q*2 = 0.00004, *Q*3 = 0.00016) with filtration compared to (*Q*1 = 0.00002, *Q*2 = 0.00006, *Q*3 = 0.00082) without filtration. See the supplement for more plots on the other datasets.

In conclusion, we described a method that results in a simple, fast and scalable tool for taxonomic profiling and abundance estimation which utilizes coverage information of individual genomes to filter out those that are unlikely to be in the sample. This is done by discarding genomes with relatively low coverage percentage by uniquely mapped reads and mapping reads in general. These simple yet important filtration steps allow SLIMM to be capable of identifying organisms with high recall rate while remaining precise. We showed that SLIMM methodology resulted in more accurate taxonomic profiling as well as predicting the individual abundance of member organisms more accurately than the other tools. We evaluated the accuracy of SLIMM against Kraken, GOTTCHA and mOTUs using 18 different datasets from multiple sources. The results show that SLIMM is superior in detecting the correct member organisms of a microbial community. SLIMM exhibited the highest F1-Score in 17 out of the 18 cases. The average F1-score across the datasets is 0.93 for SLIMM, 0.77 for Kraken, 0.54 for GOTTCHA and 0.49 for mOTUs. We have also evaluated the correctness of individual abundances using average absolute difference of predicted abundance from the true abundance. SLIMM has the lowest average absolute difference (0.00073) of all the other methods and the next best tool in this regard is Kraken with average absolute difference of 0.00116. Regarding runtime and memory consumption, SLIMM is the fastest tool, without the preprocessing step, while using significantly less memory. These advances on taxonomic profiling of microbial communities will help determine the alpha (community level) diversity and beta diversity across multiple microbial communities more reliably. This in-turn better facilitate follow-up studies such as the impacts of antibiotic usage on microbial communities and consequently on the host’s health.

##  Supplemental Information

10.7717/peerj.3138/supp-1 Figure S1 Precision—call Curves: SLIMM vs Existing Methods of 8 different datasets True Positive Rate (TPR)/recall drawn against precision. SLIMM received the highest performance for all of the datasets by detecting most of the microorganisms in each sample while staying precise.

10.7717/peerj.3138/supp-2 Figure S2 Precision—Recall Curves of different SLIMM variants for 8 different datasets True Positive Rate (TPR)/recall drawn against precision. These plots show the accuracy performance of different SLIMM variants, i.e., SLIMM, SLIMM-DG (with digital normalization), SLIMM-NF (without filtration step based on coverage landscape), SLIMM-NF-DG (without filtration but with digital normalization) and SLIMM using alignment produced by the read mapper Bowtie2. The comparison is done across 8 different datasets. SLIMM’s filtration step produced the highest performance for all of the datasets.

10.7717/peerj.3138/supp-3 Figure S3 Violin Plots of the difference between real and predicted abundances: SLIMM vs. Existing Methods The violin plots show how well the different tools predicted the abundances compared to the actual abundances across eight different datasets. From the plots, we can clearly see that SLIMM has the lowest divergence from the actual abundance for most of the samples.

10.7717/peerj.3138/supp-4 Figure S4 Violin Plots of the difference between real and predicted abundances: different SLIMM variants The violin plots show how well the different variants of SLIMM predicted the abundances compared to the actual abundances across eight different datasets.

10.7717/peerj.3138/supp-5 Figure S5 Scatter plots showing predicted vs real abundances Abundances of 8 different samples predicted by different tools compared to the true abundance used for simulation. SLIMM predicted the abundances more accurately than the other tools.

10.7717/peerj.3138/supp-6 Figure S6 Scatter plots showing predicted vs real abundances by different SLIMM variants Abundances of 8 different samples predicted by different flavors of SLIMM compared to the true abundance used for simulation.

10.7717/peerj.3138/supp-7 Data S1 Supplementary data containing, details of datasets used for this study, accuracy comparison of different methods per datasets, runtime and memory consumption of each method for individual datasets and statistical details (STDDEV, MEAN, Variance, Q1, Q2(median), Q3) of the differences b/n real and predicted abundances.

## Additional Information and Declarations

### Data Availability

The following information was supplied regarding data availability: SLIMM was developed in C++ with SeqAn Library (Döring et al., 2008). The program is available for free at https://github.com/seqan/slimm.
