# Supplementary figures and images for: SLIMM: species level identification of microorganisms from metagenomes
Source: PeerJ. 2017 Mar 28;5:e3138. doi: 10.7717/peerj.3138 (PMC5372838; doi:10.7717/peerj.3138)

A.

AB-64

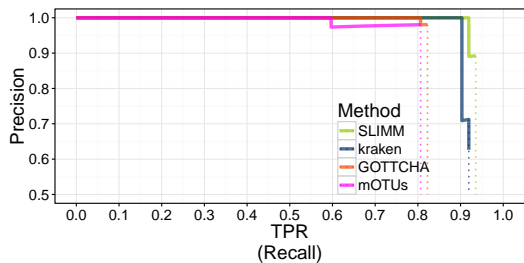

B.

HMP-mock-ill-even

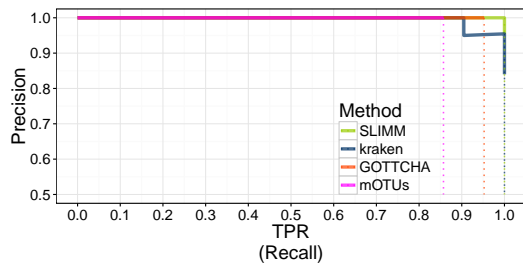

C.

HMP-mock-ill-stag

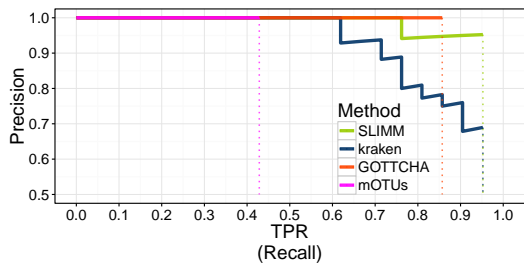

D.

random-0500-1000

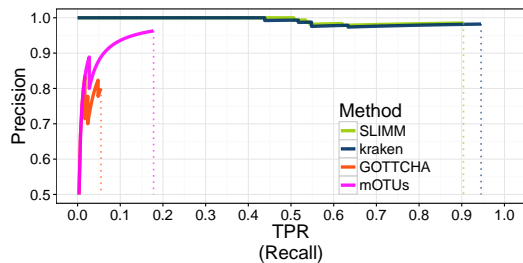

E.

CAMI M1-S001

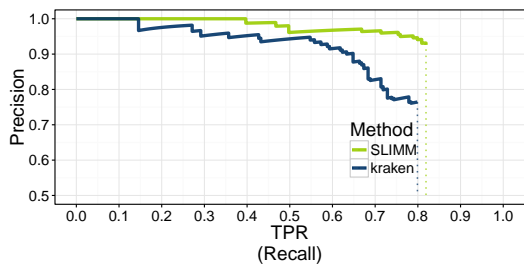

F.

CAMI M1-S002

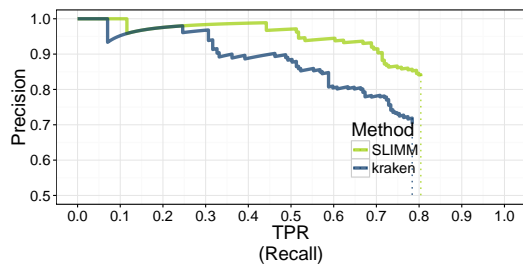

G.

CAMI M2-S001

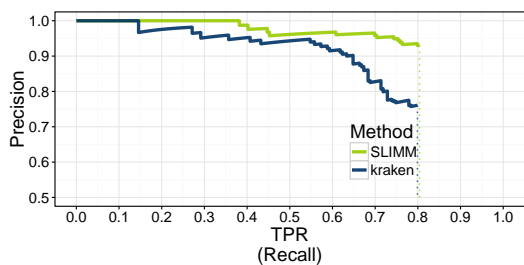

H.

CAMI M2-S002

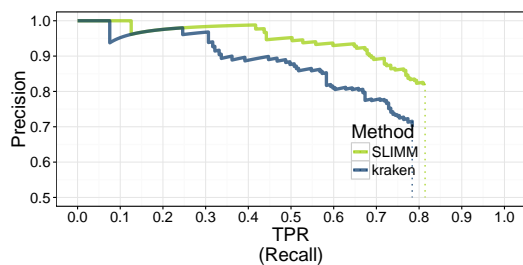

Supplement: Figure S1 — True Positive Rate (TPR)/recall drawn against precision. SLIMM received the highest performance for all of the datasets by detecting most of the microorganisms in each sample while staying precise. [file peerj-05-3138-s001.pdf]

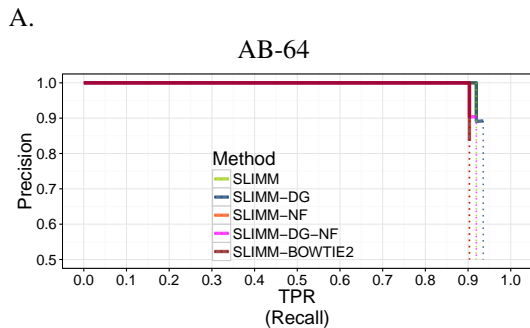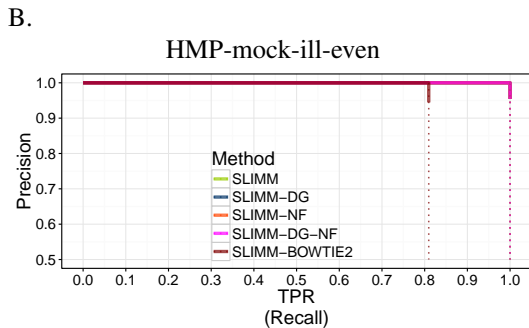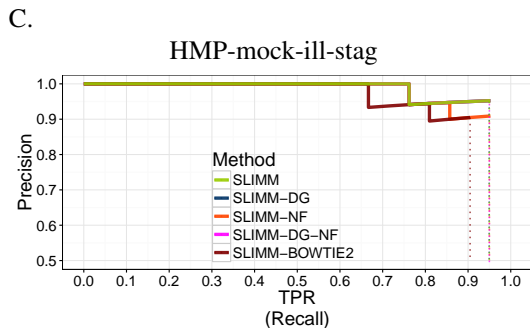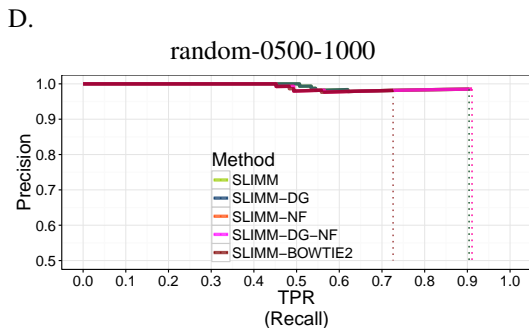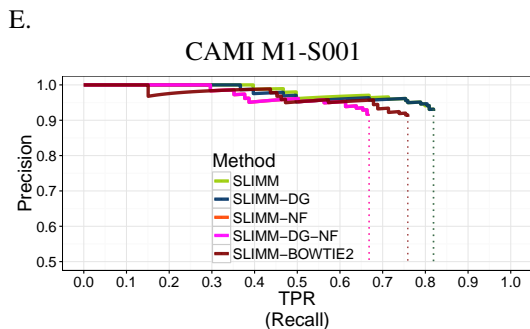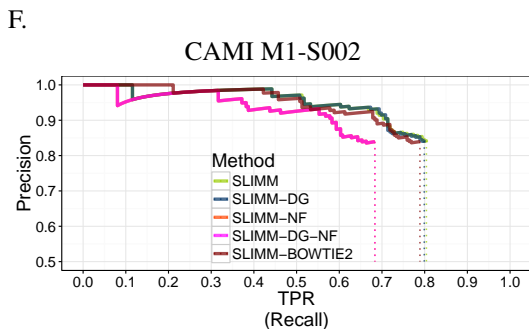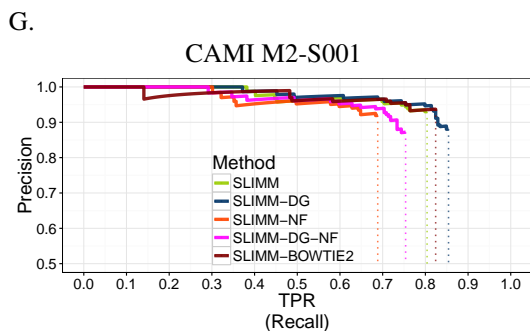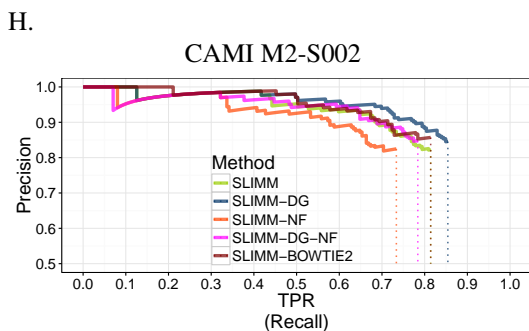

Supplement: Figure S2 — True Positive Rate (TPR)/recall drawn against precision. These plots show the accuracy performance of different SLIMM variants, i.e., SLIMM, SLIMM-DG (with digital normalization), SLIMM-NF (without filtration step based on coverage landscape), SLIMM-NF-DG (without filtration but with digital normalization) and SLIMM using alignment produced by the read mapper Bowtie2. The comparison is done across 8 different datasets. SLIMM’s filtration step produced the highest performance for all of the datasets. [file peerj-05-3138-s002.pdf]

A.

## AB-64

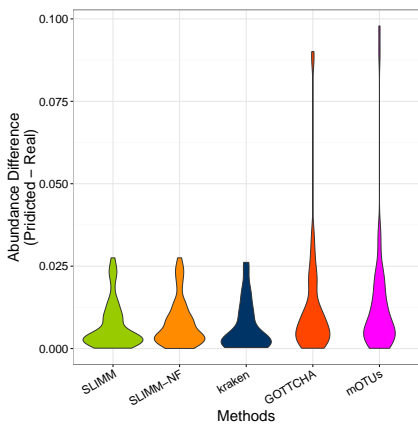

B.

## HMP-mock-ill-even

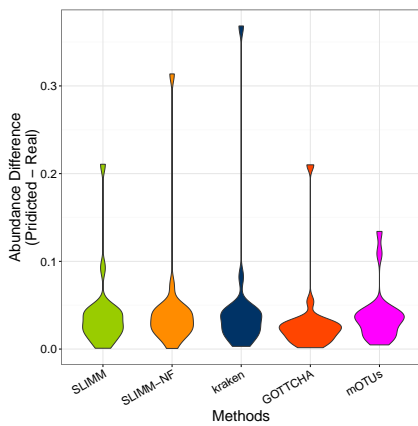

C.

## HMP-mock-ill-stag

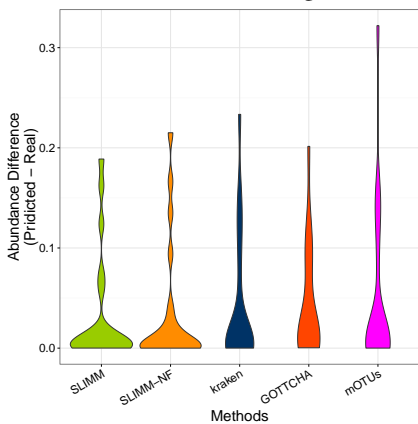

D.

## random-0500-1000

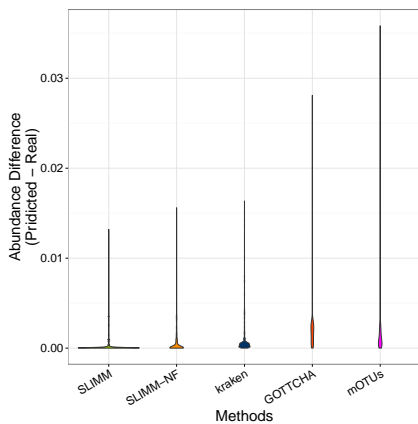

E.

## CAMI M1-S001

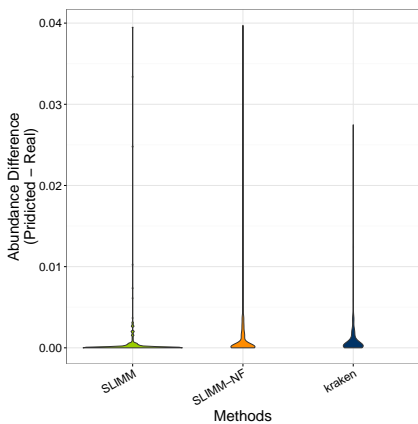

F.

## CAMI M1-S002

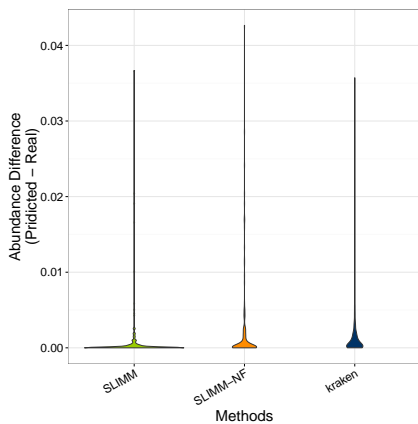

G.

## CAMI M2-S001

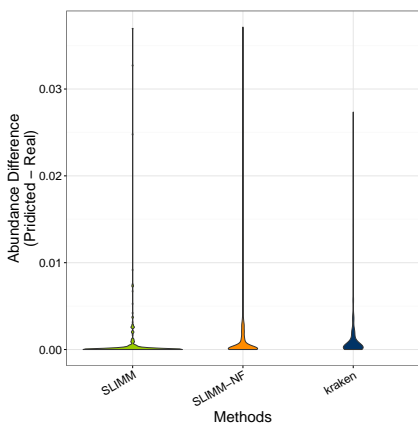

H.

## CAMI M2-S002

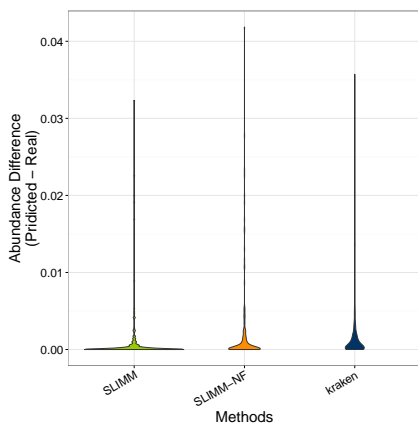

Supplement: Figure S3 — The violin plots show how well the different tools predicted the abundances compared to the actual abundances across eight different datasets. From the plots, we can clearly see that SLIMM has the lowest divergence from the actual abundance for most of the samples. [file peerj-05-3138-s003.pdf]

A.

AB-64

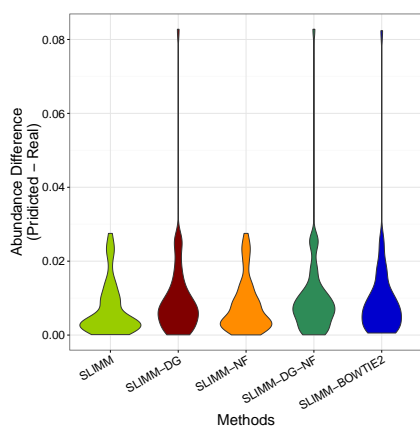

B.

HMP-mock-ill-even

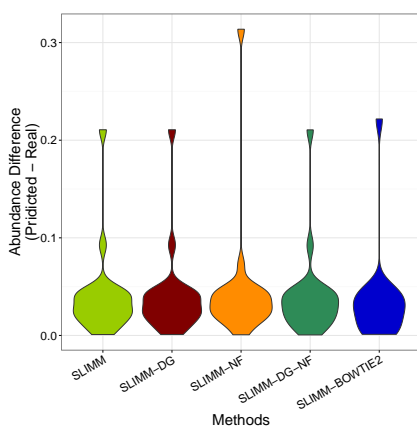

C.

HMP-mock-ill-stag

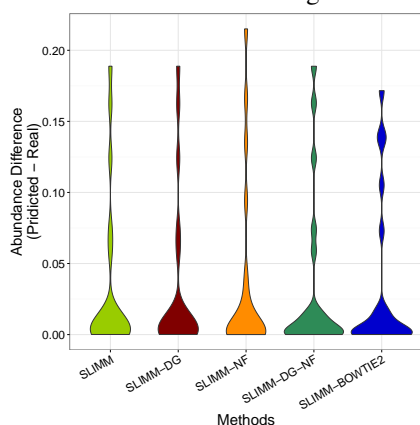

D.

random-0500-1000

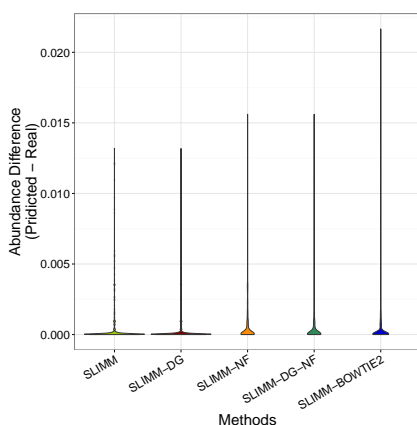

E.

CAMI M1-S001

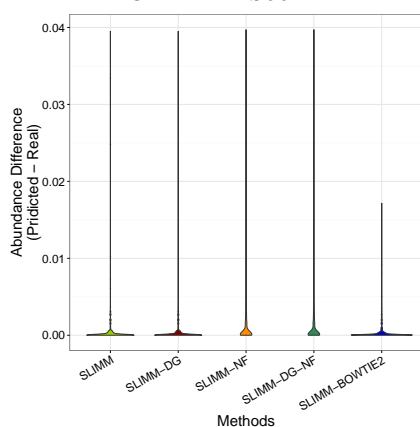

F.

CAMI M1-S002

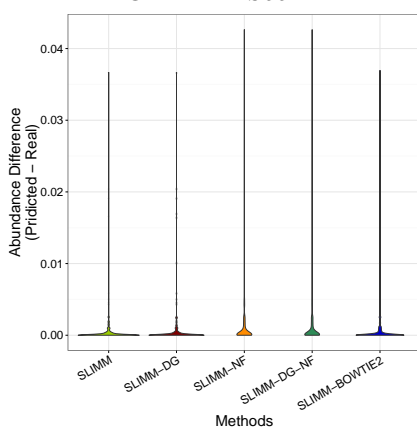

G.

CAMI M2-S001

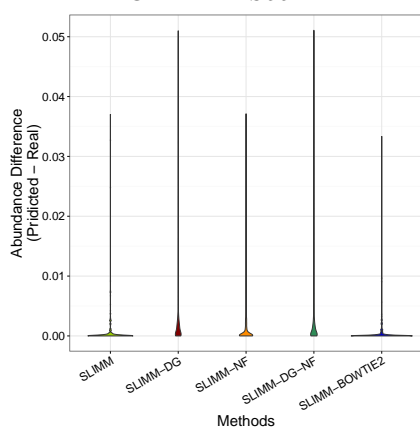

H.

CAMI M2-S002

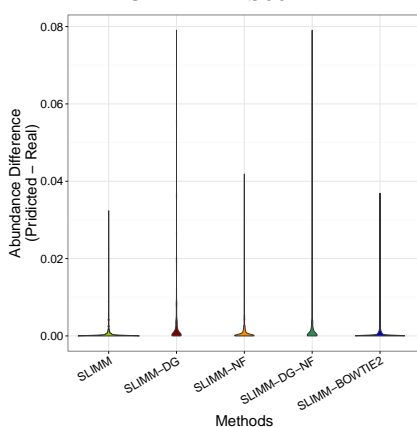

Supplement: Figure S4 — The violin plots show how well the different variants of SLIMM predicted the abundances compared to the actual abundances across eight different datasets. [file peerj-05-3138-s004.pdf]

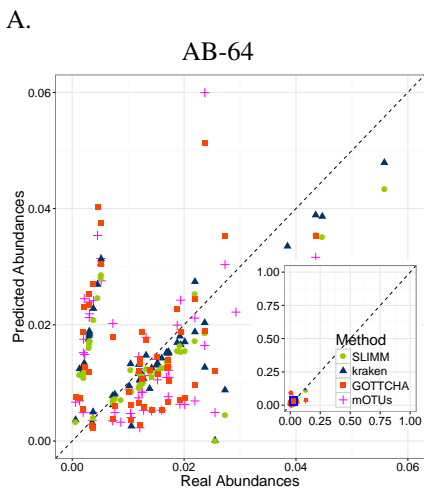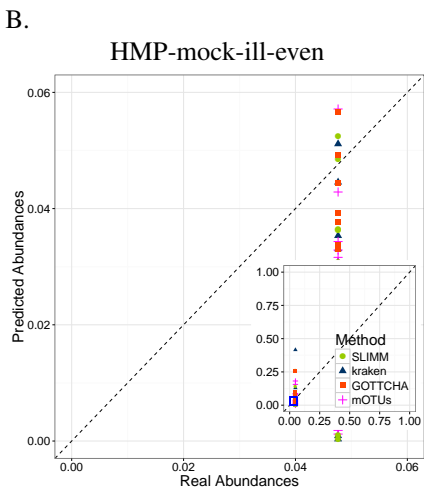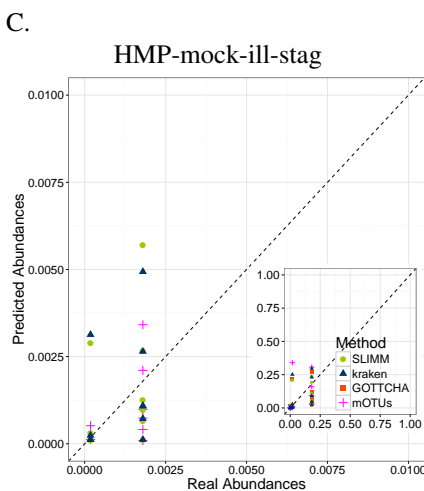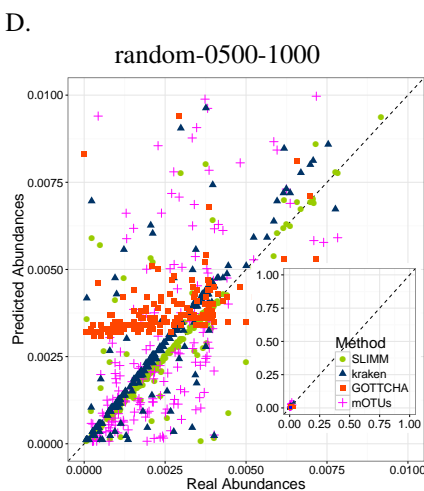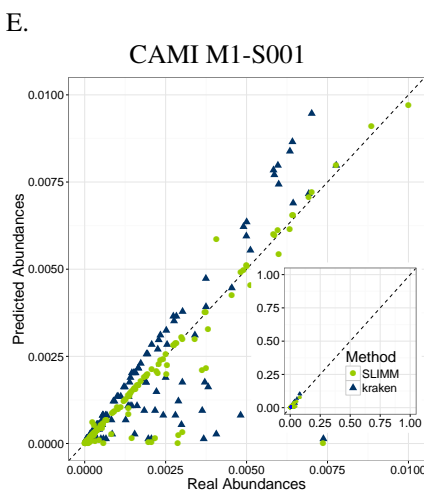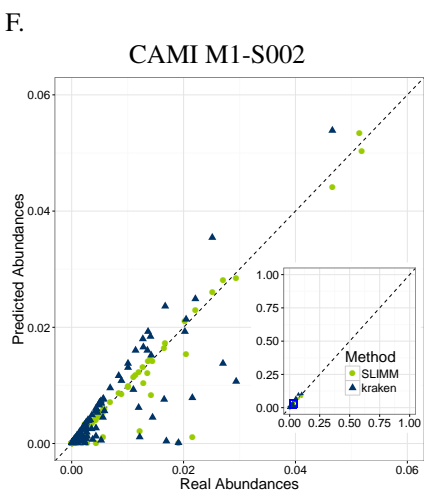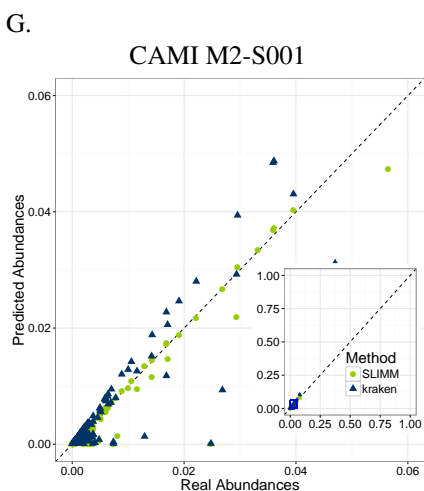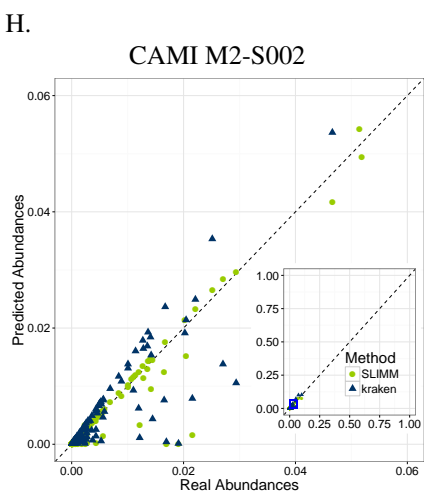

Supplement: Figure S5 — Abundances of 8 different samples predicted by different tools compared to the true abundance used for simulation. SLIMM predicted the abundances more accurately than the other tools. [file peerj-05-3138-s005.pdf]

A.

AB-64

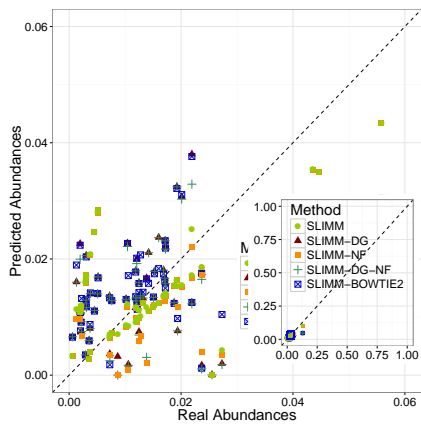

B.

HMP-mock-ill-even

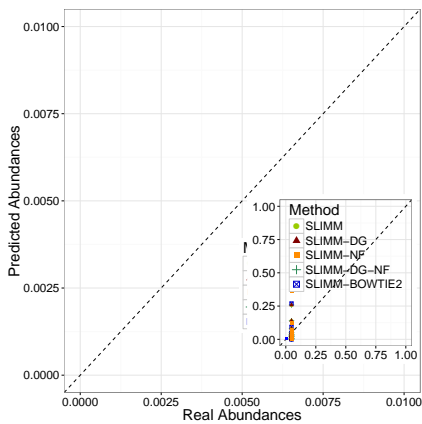

C.

HMP-mock-ill-stag

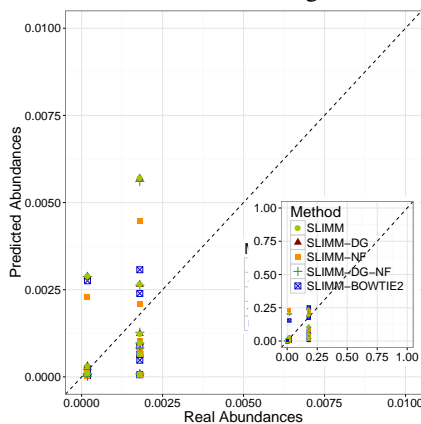

D.

random-0500-1000

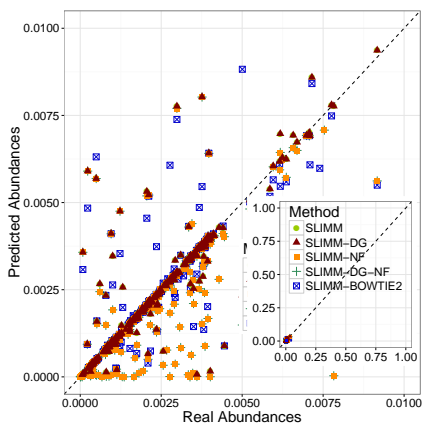

E.

CAMI M1-S001

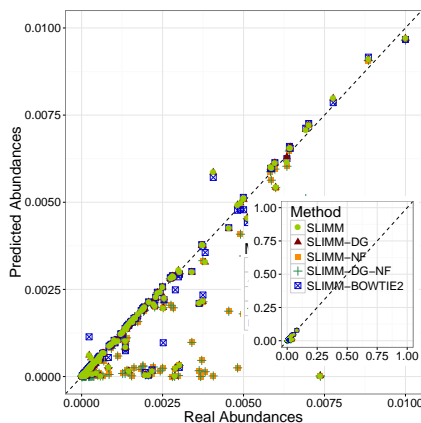

F.

CAMI M1-S002

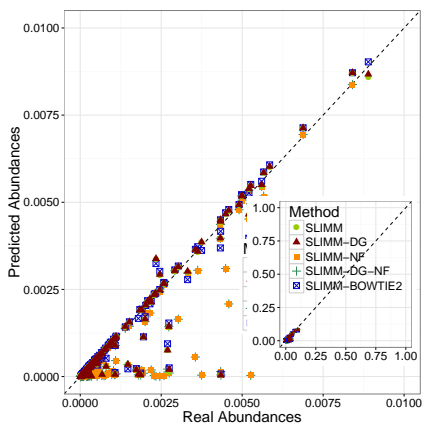

G.

CAMI M2-S001

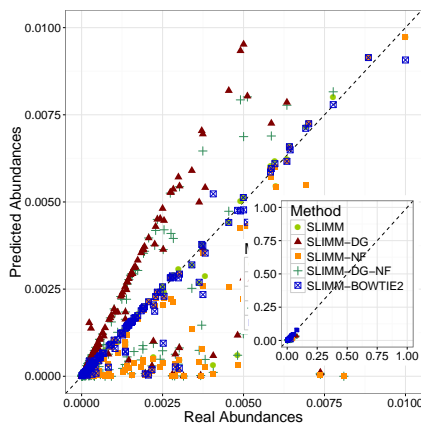

H.

CAMI M2-S002

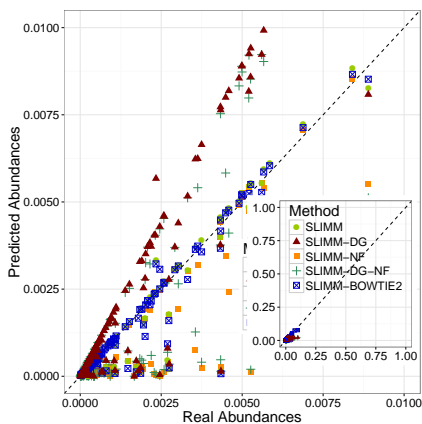

Supplement: Figure S6 — Abundances of 8 different samples predicted by different flavors of SLIMM compared to the true abundance used for simulation. [file peerj-05-3138-s006.pdf]
